# Differential modulation of NREM sleep regulation and EEG topography by chronic sleep restriction in mice

**DOI:** 10.1038/s41598-019-54790-y

**Published:** 2020-01-10

**Authors:** Bowon Kim, Eunjin Hwang, Robert E. Strecker, Jee Hyun Choi, Youngsoo Kim

**Affiliations:** 1 Korea Institute of Science and Technology, Center for Neuroscience, Seoul, South Korea; 2 Yonsei University, Medical Science Department, Seoul, South Korea; 3 University of Science and Technology, Department of Neuroscience, Daejon, South Korea; 4 VA Boston Healthcare System, Research Service and Harvard Medical School, Department of Psychiatry, Brockton, MA USA

**Keywords:** Sleep deprivation, Non-REM sleep

## Abstract

Compensatory elevation in NREM sleep EEG delta power has been typically observed following prolonged wakefulness and widely used as a sleep homeostasis indicator. However, recent evidence in human and rodent chronic sleep restriction (CSR) studies suggests that NREM delta power is not progressively increased despite of accumulated sleep loss over days. In addition, there has been little progress in understanding how sleep EEG in different brain regions responds to CSR. Using novel high-density EEG electrode arrays in the mouse model of CSR where mice underwent 18-h sleep deprivation per day for 5 consecutive days, we performed an extensive analysis of topographical NREM sleep EEG responses to the CSR condition, including period-amplitude analysis of individual slow waves. As previously reported in our analysis of REM sleep responses, we found different patterns of changes: (i) progressive decrease in NREM sleep duration and consolidation, (ii) persistent enhancement in NREM delta power especially in the frontal and parietal regions, and (iii) progressive increases in individual slow wave slope and frontal fast oscillation power. These results suggest that multiple sleep-wake regulatory systems exist in a brain region-specific manner, which can be modulated independently, especially in the CSR condition.

## Introduction

Despite the fact that most people are suffering from daily insufficient sleep rather than a single event of sleep deprivation, most sleep studies have used several hours to one day of total sleep deprivation to infer the sleep responses to chronic sleep restriction (CSR). The most widely used electrophysiological measure of sleep intensity or depth is the spectral power of EEG delta waves (4 Hz or less) in NREM sleep, alternatively called “slow wave activity^1^”. After an event of prolonged spontaneous wakefulness or sleep deprivation, typical homeostatic sleep responses include compensatory increases in sleep duration and/or intensity in the following sleep. Using different rodent models of CSR, we have shown that when rats are sleep restricted for 5 consecutive days, homeostatic increases in sleep duration and intensity are progressively reduced from day 1 to 5^2,3^. This pattern of altered homeostatic sleep responses has also been observed in other rat CSR studies^4–10^. But other studies have shown maintaining elevated levels in NREM delta power during CSR^11^. To our knowledge, however, no studies have reported progressive increase in NREM delta power as sleep restriction (SR) is repeated despite of accumulating net sleep loss over days.

Recent findings indicate that sleep-correlated EEG changes may not occur uniformly throughout the cerebral cortex^12,13^. Consistent with these observations, a previous rat CSR study reported persistently elevated level of NREM delta power in the frontal cortex but a diminishing NREM delta power in the occipital cortex^11^. Although high-density EEG (HD-EEG) methods are being widely used in humans, current rodent EEG methods using conventional screw electrodes typically sample 3 or fewer cortical areas, making it difficult to analyze dynamic changes of EEG signals in the whole cortical regions. To end this, we developed a novel 40-channel high-density electrode array which can monitor EEG signals from almost entire cortical areas of mice. Recently, using both conventional screw electrodes and HD-EEG electrode array with the mouse model of CSR, we reported 2 distinct patterns of REM sleep responses; persistent increases in global slow oscillation and progressive increases in local fast oscillation over 5 sleep restriction (SR) days^14^.

The power spectral analysis of NREM delta power is typically assessed using a fast Fourier transform (FFT). However, this method has limitations due to its averaging process over a given time window of data. For example, power spectral analysis does not separately report the amplitude and incidence of waves of interest^15^. Therefore, it cannot differentiate low-amplitude, high-incidence waves from high-amplitude, low-incidence waves^16^. The period-amplitude analysis method detects individual waves of interest from continuous EEG signals and reports wave period, amplitude and incidence components separately^15^. However, the period-amplitude analysis typically requires a band-pass filtering^17^, which may distort the original wave forms^15^. To overcome the limitations of the power spectral analysis and period-amplitude analysis, in this study we performed an extensive analysis of topographical NREM sleep EEG responses to the CSR condition, using both period-amplitude analysis of individual slow waves and power spectral analysis. Similar to our findings in REM sleep^14^, here we report brain region-specific multiple patterns of changes in NREM sleep regulation in response to CSR, which has not been very evident in previous acute sleep deprivation studies.

## Results

As depicted in our previous paper (Fig. S2)^14^, 24-h baseline (BL) sleep recording was started 6 h after the light onset (*zeitgeber* time (ZT) 6) on the fourth day. Next, 18-h sleep deprivation (ZT6–24) followed by 6-h sleep oppertunity (SO) (ZT0–6) was repeated for 5 consecutive days. After 5 d of SR, mice were given 3 d of 24-h unrestricted recovery sleep (R) period. Conventional screw EEG data were recorded continuously for all 9 experimental days, while HD-EEG data was recorded for only ZT0–3 of each day (i.e., the first 3 h of the SO).

### Progressive reduction in NREM sleep time during the daily 6-h SO

**Figure 1:**
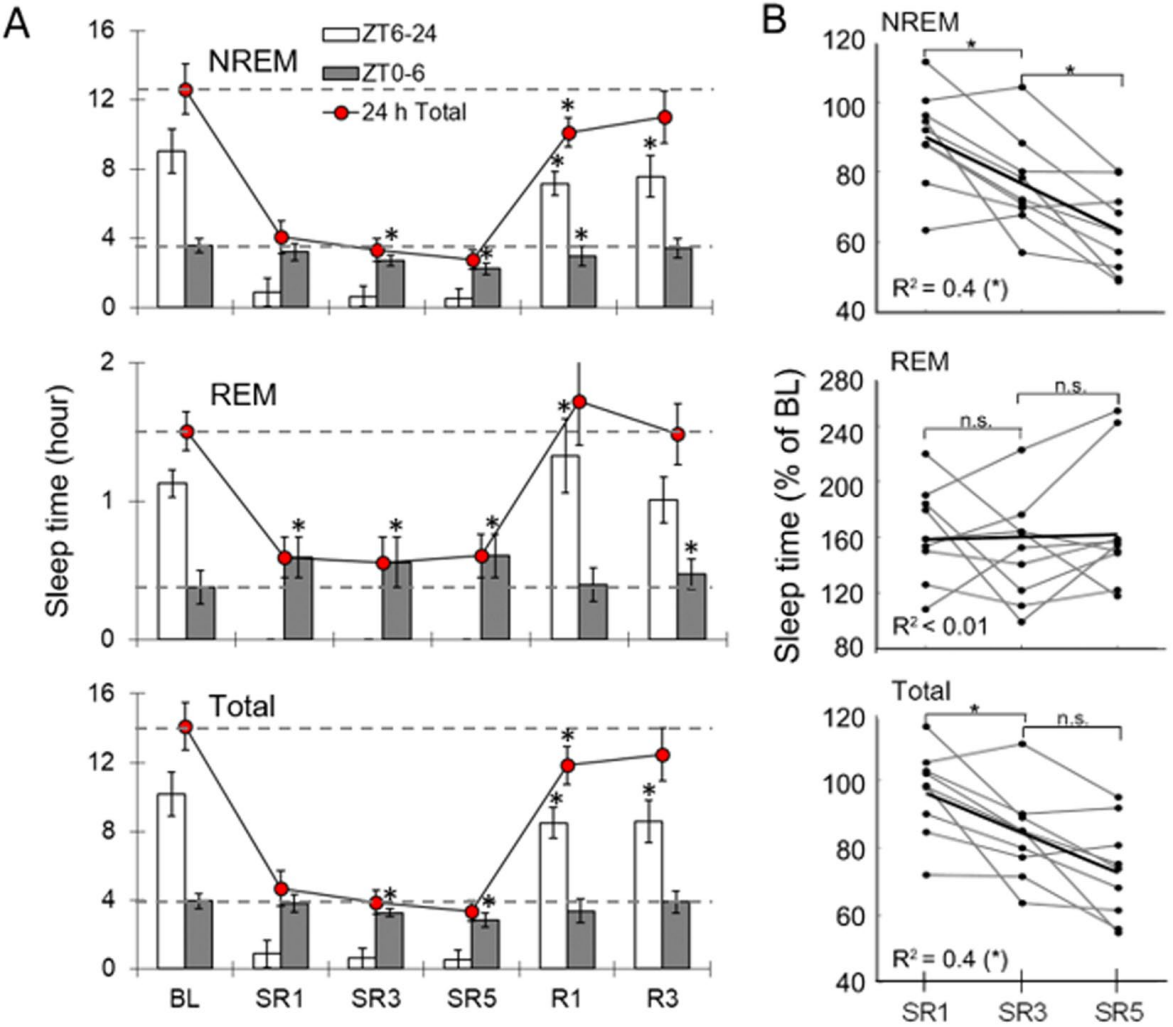
NREM sleep time during the daily 6-h sleep opportunities was progressively decreased during chronic sleep restriction. (**A**) The amounts of NREM (top), REM (middle), and total (bottom) sleep time during baseline (BL), sleep restriction (SR1-SR5) and recovery sleep (R1-R3) days were determined across 18-h (zeitgeber time (ZT) 6–24; open bar; sleep deprivation period on SR days), 6-h (ZT0–6; grey bar; period sleep allowed) and 24-h (ZT0–24, filled circle) time blocks. All comparisons were made to the corresponding BL (repeated measure of ANOVA; *n* = 9), and the asterisk (*) indicates statistical significance (*p* < 0.05). All values represent mean ± SD. (**B**) Linear regression analysis for changes in relative sleep durations on SR days expressed as a percentage of BL levels. R^2^ values indicate Pearson’s correlation coefficients and the asterisk (*) indicates statistical significance (*p* < 0.05).

On the BL day, mice spent 12.6 ± 1.4 h in NREM sleep over the 24-h period, derived from 9.0 ± 1.3 h in the ZT6–24 time block (18 h) and 3.6 ± 0.4 h in the ZT0–6 time block (6 h) (Fig. 1A top panel). The sleep deprivation procedure produced >95% wakefulness during the daily 18-h sleep deprivation periods (ZT6–24) on SR days; specifically, mice slept in the wheel less than 0.9 ± 0.8 h on SR1 and 0.5 ± 0.5 h on SR5 (Fig. 1A bottom panel) with complete absence of REM sleep (Fig. 1A middle panel). Despite the presumably continuous accumulation of sleep loss over 5 SR days, the NREM sleep time during 6-h SO (ZT0–6) periods decreased progressively from SR day 1 to 5 (Fig. 1A top panel; *F*(5, 40) = 9.621, *p* < 0.001; SR1: −10.5%, *p* = 0.065; SR3: −24.5%, *p* = 0.001; SR5: −37.4%, *p* < 0.001) in a linear fashion (Fig. 1B top panel), compared to the corresponding time period of the BL day. Following the 5 days of SR, mice had 3 days of unrestricted SO. Even on recovery sleep day 3, total NREM sleep time over the 24-h period was still below the BL level (Fig. 1A. *F*(5, 40) = 150.434, *p* < 0.001; R1: −19.8%, *p* = 0.004; R3: −12.8%, *p* = 0.058).

### Stable REM sleep homeostasis during CSR

Changes in REM sleep time over experimental days showed a different pattern from that of total sleep time and NREM sleep time (Fig. 1A middle panel). On the BL day, mice spent an average of 1.50 ± 0.14 h in REM sleep over the 24-h period, derived from 1.13 ± 0.10 h during the ZT6–24 time block and 0.38 ± 0.12 h during the ZT0–6 time block. During the 18-h sleep deprivation (ZT6–24) on SR days, REM sleep was completely absent. During the daily 6-h SO (ZT0–6), REM sleep time was significantly increased on SR days 1, 3 and 5 (*F*(5, 40) = 10.776, *p* < 0.001; post-hoc all *p* ≤ 0.002), in contrast to the negative rebound of NREM sleep time (Fig. 1A,B middle panel). However, the magnitude of this gain (+0.22 h on SR1 and SR5 to + 0.18 h on SR3 relative to baseline) was far less than the amount of REM sleep lost each day of the CSR protocol (approximately 1.1 h of REM sleep lost per day). On recovery sleep day 1, the rebound of REM sleep over the 24-h period was not significant (*F*(5, 40) = 120.996, *p* < 0.001; post hoc *p* = 0.057), which contrasts with the significantly reduced NREM sleep time on R1 (Fig. 1A top panel).

### Progressive reduction in total sleep time during the daily 6-h SO without complete recovery even after 3 days of unrestricted sleep

The pattern of changes in total sleep time (Fig. 1A bottom panel) was very similar to that of NREM sleep time changes (Fig. 1A top panel). On the BL day, total sleep time was 14.1 ± 1.4 h over the 24-h period, derived from 10.2 ± 1.3 h in the ZT6–24 time block and 4.0 ± 0.4 h in the ZT0–6 time block (Fig. 1A bottom panel, BL). Compared to the corresponding time period of the BL day, reductions in the total sleep time during the 6-h SO (ZT0–6) periods (*F*(5, 40) = 6.703, *p* < 0.001) were significant on SR3 (−17.6%, *p* = 0.006) and SR5 (−28.2%, *p* < 0.001) and the trend was linear (Fig. 1B bottom panel). During recovery sleep days, total sleep time over the 24-h period (*F*(5, 40) = 166.273, *p* < 0.001) was still below the BL level (R1: −2.3 h, *p* = 0.010; R3: −1.64 h, *p* = 0.064).

### Cumulative reduction in NREM sleep consolidation during the daily 6-h SO

**Figure 2:**
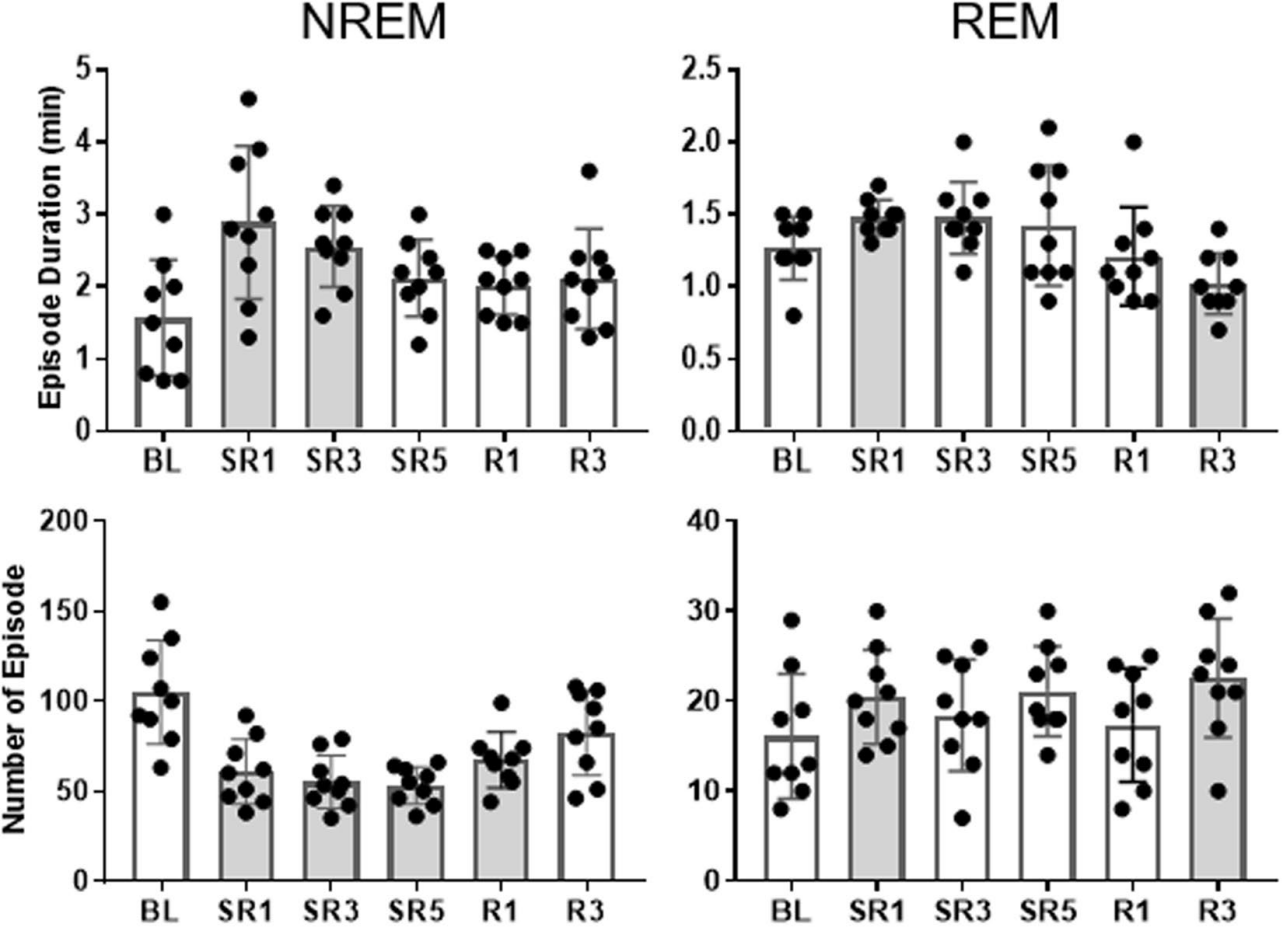
The compensatory increase in NREM sleep consolidation on SR1 was progressively reduced throughout the rest of SR days. NREM **(left panel)** and REM **(right panel)** sleep episode duration (top panel) and number (bottom panel) at ZT 0–6 time blocks (mean ± SD) were analyzed. All comparisons were made to the BL (repeated measure ANOVA; *n* = 9), and the shaded bars indicate statistical significance (*p* < 0.05).

Sleep consolidation is one of the most important measures of sleep homeostasis^18,19^. To determine whether mice exhibit increased sleep consolidation, NREM sleep episode duration and number were measured during the 6-h SO (Fig. 2 left panel). A significant time effect was detected in NREM episode duration (*F*(5, 40) = 4.977, *p* = 0.001) and number (*F*(5, 40) = 11.218, *p* < 0.001). NREM episode duration was increased substantially after 18-h sleep deprivation on SR1 (2.89 ± 1.06 min vs. 1.57 ± 0.80 min, *p* = 0.002). However, the magnitude of the increase was gradually reduced as SR was repeated (e.g., 2.56 ± 0.55 min on SR3, *p* = 0.005), eventually becoming non-significant on SR5 (2.11 ± 0.53 min, *p* *=* 0.13). Consequently, there was a trend of decline in NREM sleep episode duration from SR1 to SR5 (*P* = 0.094). In contrast, the number of NREM episodes remained significantly lower than the baseline level on SR days (all *p* < 0.001), and even on R1 (67.3 ± 15.3, *p* = 0.013).

As shown in Fig. 2 (right panels), a significant time effect was also detected in REM episode duration (*F*(5, 40) = 4.066, *p* = 0.004) and number (*F*(5, 40) = 3.791, *p* = 0.007). REM episode became significantly longer on SR1 (1.48 ± 0.12 min, *p* = 0.018), compared to BL (1.27 ± 0.22 min). But it became significantly shorter on R3 (1.02 ± 0.21 min, *p* = 0.008). In contrast, the number of REM episode remained at elevated levels throughout experimental days reaching statistical significance on SR1 (20.4 ± 5.2, *p* = 0.003) and R3 (22.6 ± 6.6, *p* = 0.004) compared to BL (16.1 ± 7.0).

### NREM delta power did not increase during the daily 6-h SO throughout SR days

**Figure 3:**
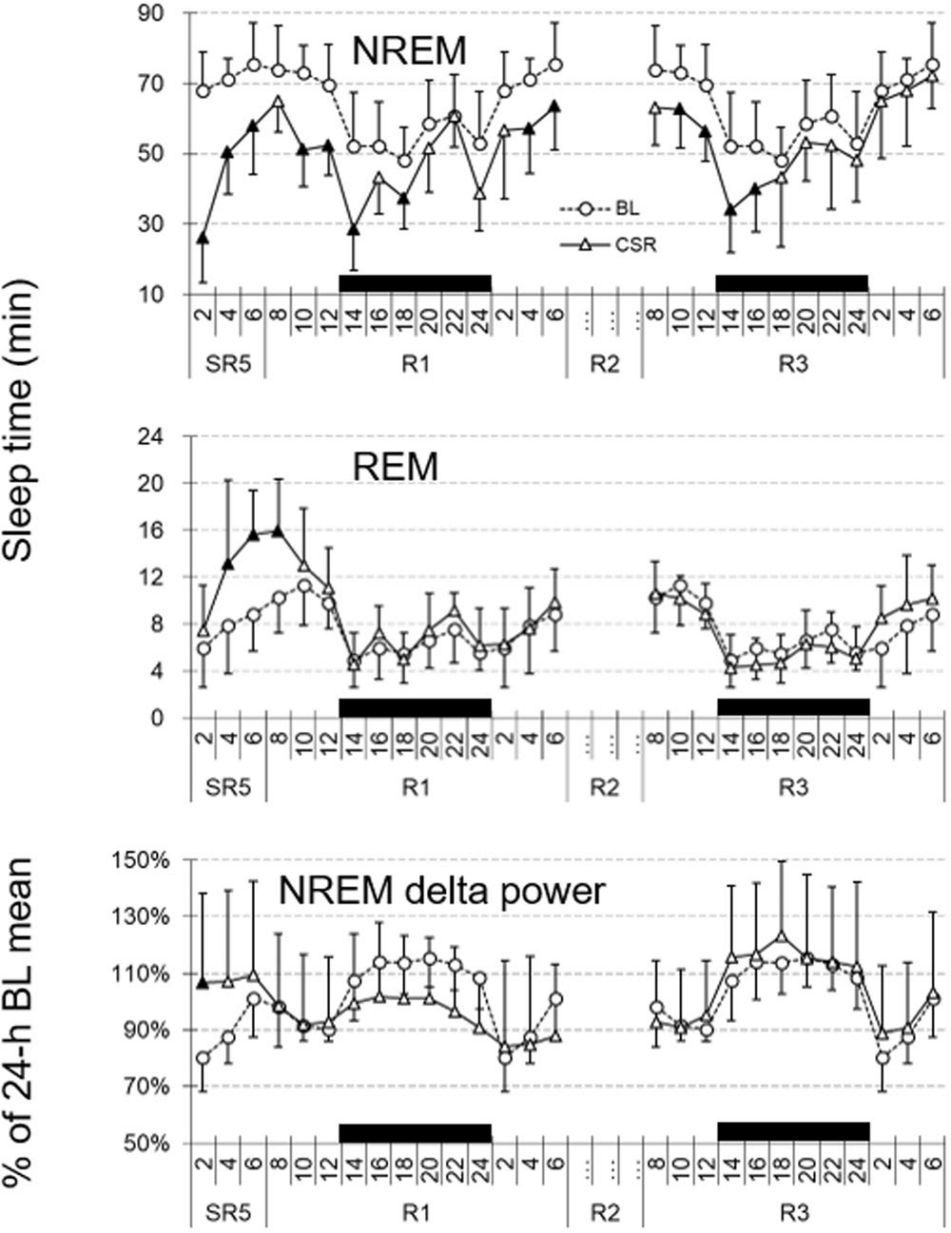
Reduced NREM sleep time persisted on recovery sleep days. Sleep time and NREM delta power were depicted for the 24-h BL (circles) and the 3 recovery days (triangles) in 2-h intervals. Note that the 24-h BL distribution was double-plotted in order to allow visual comparisons with all recovery days. The 12-h dark periods are indicated with black bars at the bottom. The filled triangles indicate statistical significance (*p* < 0.05) compared to the corresponding baseline (paired *t*-test, *n* = 9).

**Figure 4:**
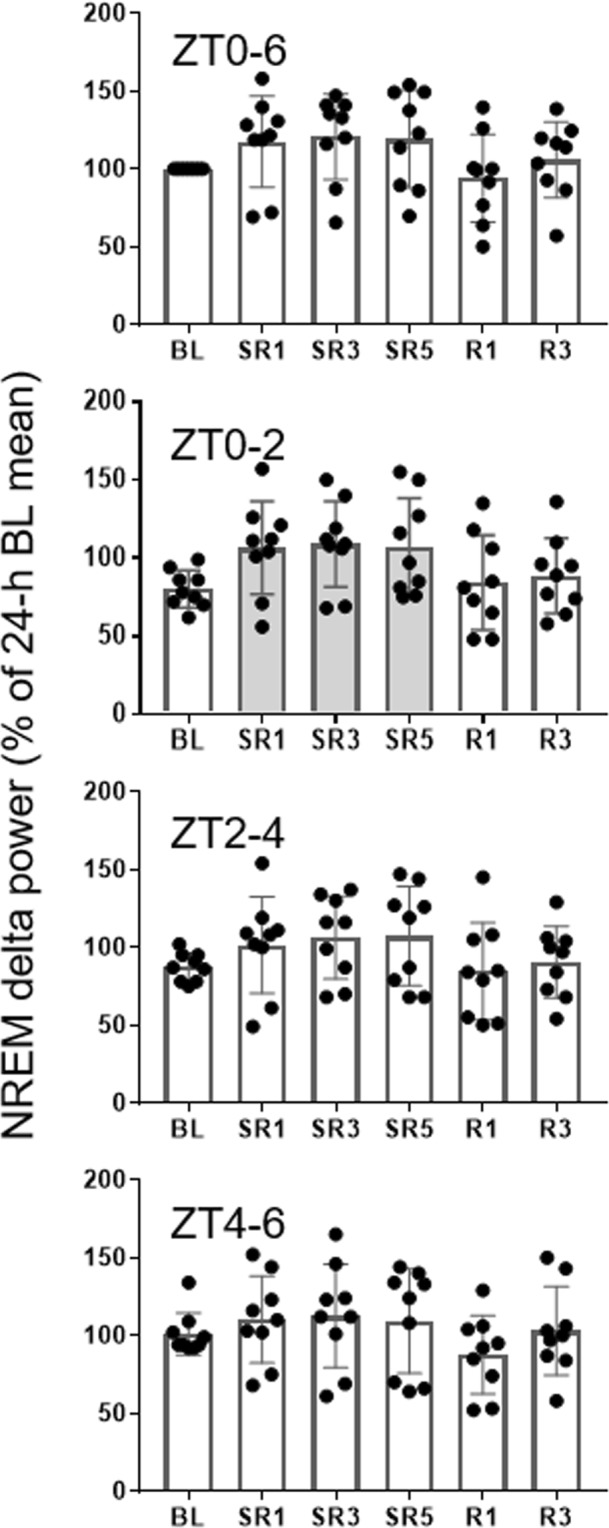
Changes in NREM delta power during the whole 6-h sleep opportunities were not significant. Delta power of 6-h bin (top panel) or 2-h bin (bottom panels) was determined across ZT0–6 time blocks (mean ± SD). Significantly elevated NREM delta power (expressed in relative change from 24 h BL mean) was observed only at the first 2 h (ZT0–2) of the 6-h sleep opportunities on SR1, SR3, and SR5 (bottom panel). The shaded bars indicate statistical significance (*p* < 0.05) compared to the corresponding BL hours (repeated measure of ANOVA; *n* = 9).

**Figure 5:**
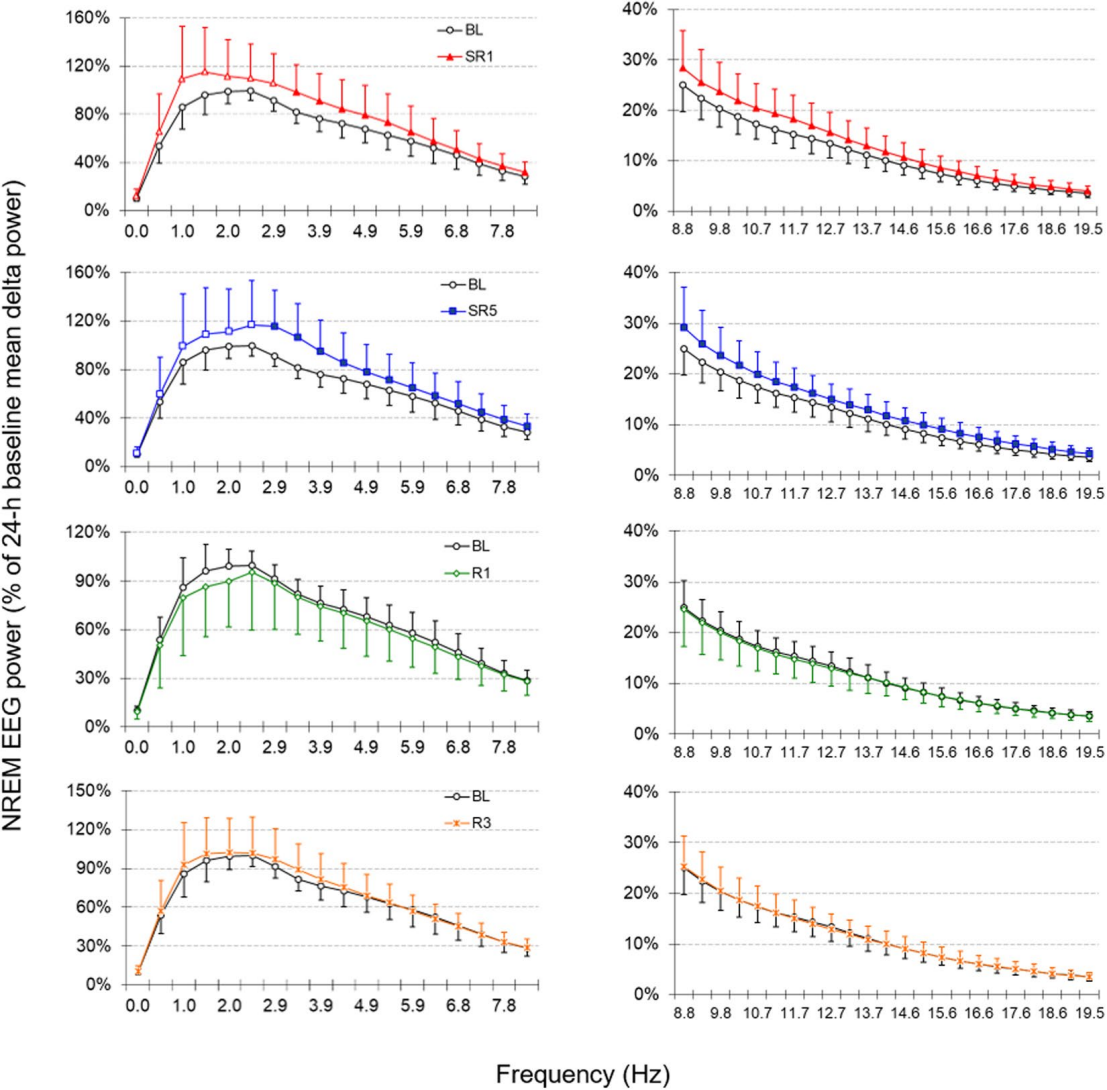
NREM EEG power during the 6-h sleep opportunities was increased at high frequency. Power spectral analysis indicates that following 18-h sleep deprivation on sleep restriction days, NREM EEG power of ZT0–6 was significantly increased at high frequency bands, including theta (4–8 Hz) and beta (12–20 Hz), on sleep restriction days (i.e., SR1 and SR5; upper panels). This increase was no longer observed on recovery sleep days (i.e., R1 and R3; lower panels). The filled symbols indicate statistical significance (*p* < 0.05) compared to the corresponding baseline (paired-t test, *n* = 9).

The sleep time data revealed that NREM sleep duration and consolidation during the daily 6-h SO decrease as the days of SR progress. Therefore, NREM EEG delta power was examined to determine whether animals compensate for their loss of sleep time by increasing sleep intensity. Interestingly, the BL of NREM delta power was low during the first 4 h of the light period and became higher for the next 4 h (Fig. 3). This time course pattern is not commonly found in other rodent or human sleep studies where NREM delta power is typically highest at the beginning of their rest period and exponentially decays as sleep continues^20^. Regardless of the low baseline, when the entire 6-h SO was analyzed, increases in NREM delta power were not statistically significant on any SR days we measured compared to the corresponding baseline values (Fig. 4 top panel; (*F*(5, 40) = 3.936, *p* = 0.05). This pattern was also evident with the power spectral analysis of NREM sleep (0~20 Hz, Fig. 5): only the power at the frequency of 3 Hz or higher was significantly elevated during SR days 1 and 5. When the 6-h SO NREM delta power was analyzed in 2-h bin, a significant time effect was detected only in the first 2-h period of 6-h SO (Fig. 4 bottom 3 panels; ZT0–2; *F*_(3, 24)_ = 5.993, *p* = 0.003; SR1, SR3 and SR5 all <0.05).

### NREM recovery sleep lasts up to 3 d after the end of 5 d of SR

The time course of NREM sleep duration in 2-h intervals during recovery sleep days 1 and 3 is shown in Fig. 3 (top panel). Reduced levels of NREM sleep persisted on R1 as well as the first half of R3 (i.e., hours 8–14), finally returning to the BL levels on the last half of R3. In contrast, REM sleep (Fig. 3 middle panel) showed robust rebound only during the light period (hours 2–8) immediately after the end of last sleep deprivation on SR5 and rapidly returned to the BL level from the dark onset of R1. NREM delta power (Fig. 3 bottom panel) showed an initial increase during the 6-h SO on SR5 followed by prolonged reduced level (also known as a “negative rebound”) on R1, even though statistically significant change (*p* = 0.019) was detected only at the first 2 h on SR5. NREM delta power completely returned to the BL level on R3.

### Persistent enhancement in NREM delta power and progressive increase in frontal fast oscillation power

**Figure 6:**
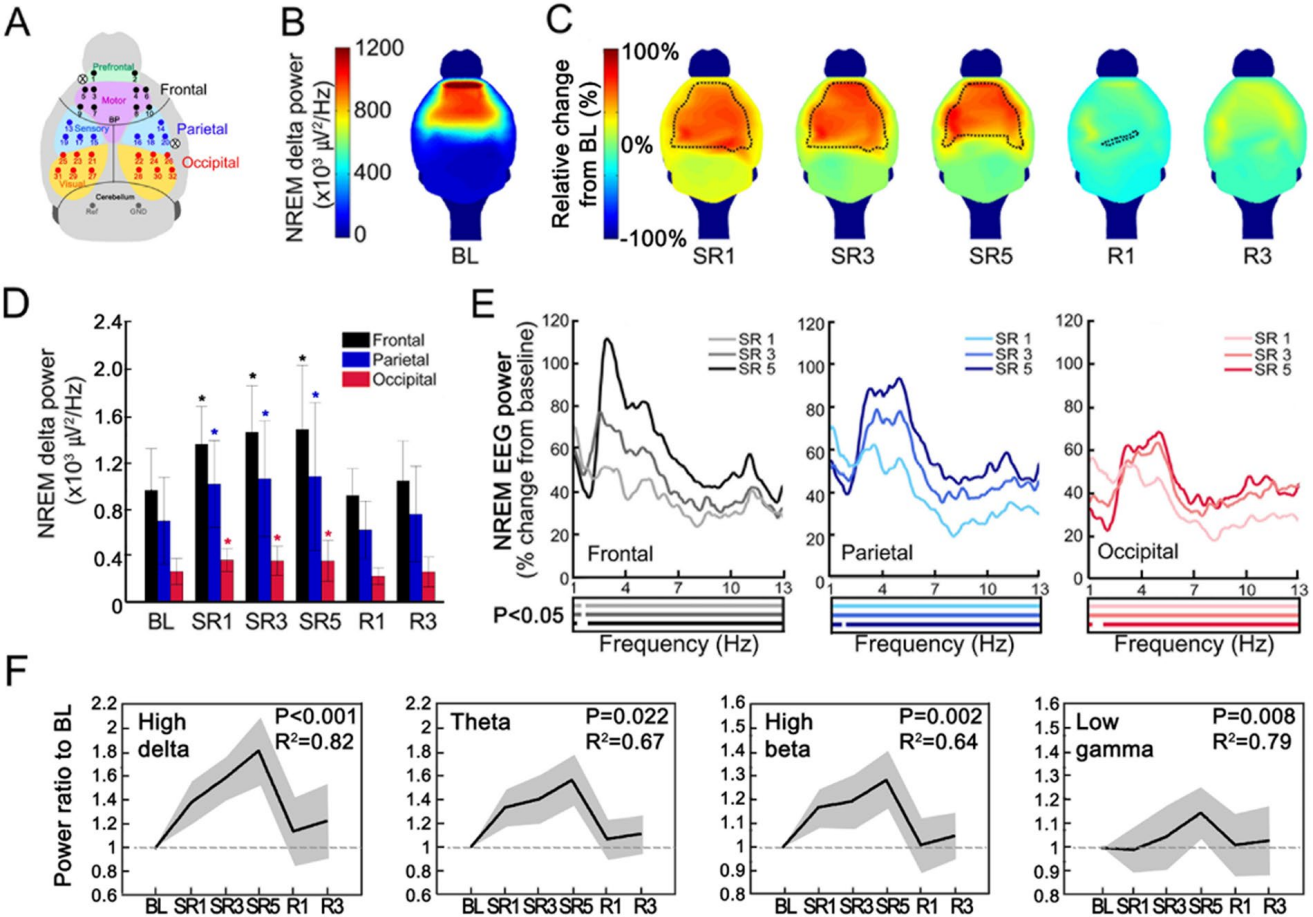
NREM high oscillation increased progressively while delta oscillation increased persistently. (**A**) Black, blue and red dots represent the electrode positions of high-density electrode array placed above the frontal, parietal, and occipital regions, respectively. White circles with X mark represent the position of screw electrodes. Black lines approximately mark the coronal and lambdoidal sutures of the skull. BP, bregma point. (**B**) Topographic maps from group averaged data of NREM delta power (1–4 Hz) during ZT1–3 on baseline day (BL; *n* = 9). Scales are as in the color bar on the left (**B**,**C**). **(C)** Topographic distribution of the mean change of NREM delta power on sleep restriction (SR) and recovery (R) days from the BL. NREM delta power was significantly enhanced mainly in the anterior half of the cortex. Dotted black lines represent the boundary of significant changes compared to the BL (*p* < 0.05, paired t-test). **(D)** Mean absolute NREM delta power (±SD) in the frontal (channel #1–10), parietal (channel #13–20) and occipital (channel #21–32) regions during ZT1–3 on experimental days. Statistically significant increases from BL delta power were observed throughout SR days. Asterisks (*) indicate statistical significance compared to BL (*p* *<* 0.05, paired t-test). **(E)** Relative changes in NREM power spectrum of 3 different brain regions from BL levels: frontal (left), parietal (middle), and occipital cortex (right). In each region, light, medium, and dark lines indicate SR1, SR3 and SR5, respectively. Statistically significant changes from BL were indicated thick lines in the box below each spectrum (*p* *<* 0.05, paired t-test). Line colors are matched between power spectrum and statistical significance box. **(F)** EEG power ratios of the frontal region (channel #1–10) to BL across SR and R days. Slopes of different EEG power bands in individual mouse were calculated by linear regression over SR days. Sign test for slopes of individual mouse was performed and *p*-values are marked in the plots with mean R-squared values for 9 mice. The SD values were depicted in grey. NREM high delta (3–4 Hz), theta (5–10 Hz), high beta (21–27 Hz) and low gamma (34–46 Hz) power showed progressive increases over SR days while overall delta power (1–4 Hz) showed persistent increases.

Regional influences of CSR on NREM delta power were analyzed from the HD-EEG data during ZT1–3 on BL, SR 1, 3, and 5 and R days 1 and 3 (Fig. 6). The absolute NREM delta power was higher in the frontal area than other cortical areas (Fig. 6B). The topography of relative power changes in the delta range (1–4 Hz) on NREM episodes showed a significant increase in most cerebral cortex regions as the mice experienced daily SR (Fig. 6C). On the recovery sleep days, none of the cortical regions showed a significant increase in delta power compared to the BL day (Fig. 6C).

Based on the report that CSR increased NREM delta power mainly in the frontal and parietal cortices^11^, NREM delta power were separately calculated by different brain regions, averaging the EEG signals for the frontal, parietal and occipital region (Fig. 6A). Regardless of the SR conditions, the frontal EEG showed higher absolute delta power compared to the other regions (*p* < 0.01, paired t-test, Fig. 6D). NREM delta power showed significant increases in all three brain regions on SR days 1, 3, and 5, compared to the BL day (Fig. 6). However, changes within the SR days (e.g., SR1 versus SR3 or SR3 versus SR5) were not statistically significant (*p* > 0.05, paired t-test).

In Fig. 6E, the daily influences of SR on the entire NREM EEG power spectrum in 3 different brain regions were analyzed as relative changes from the BL levels. Most robust increases were observed in 3~4 Hz range in the frontal region (e.g., 100 + % increase on SR5) while slower oscillation (e.g., > 2 Hz) showed a very opposite trend. Similar patterns were also observed in other brain regions with a lesser extent. Next, to further examine the change pattern of frontal EEG power on SR days, slopes of different power bands in individual mouse were calculated by linear regression (Fig. 6F). To our surprise, NREM high delta (3–4 Hz), theta (5–10 Hz), high beta (21–27 Hz)and low gamma power (34–46 Hz) showed progressive increases from SR day 1 to 5 while overall delta power (1–4 Hz) maintained at the elevated levels (Fig. 6D). This finding is consistent with the change pattern of the global NREM delta power measured by conventional EEG electrodes during the whole 6-h SO (Fig. 6 top panel).

### Progressive increase in slow wave occurrence rate, amplitude and slopes during ZT1–3 of SR days

**Figure 7:**
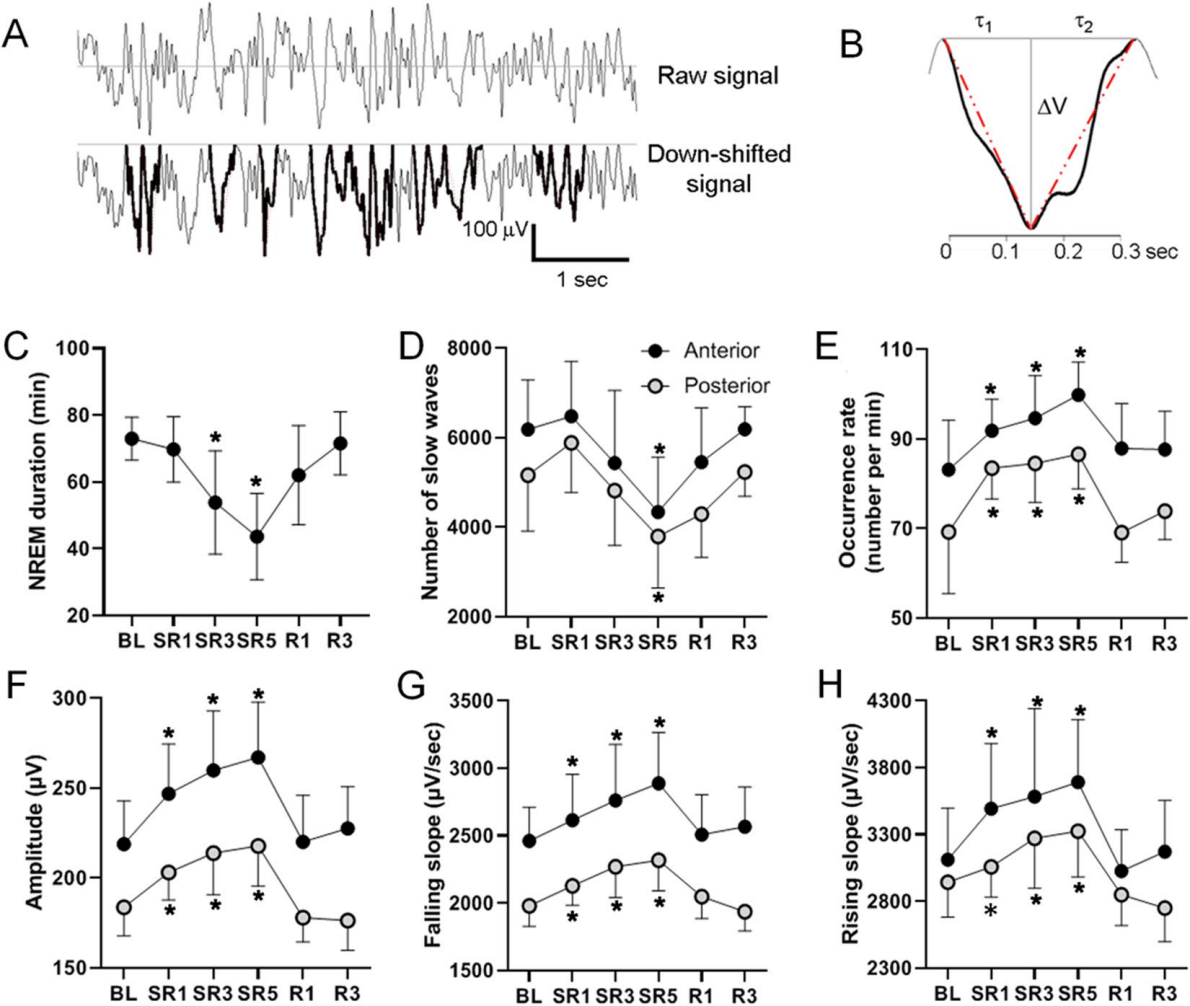
Slope of slow waves increased progressively across 5 SR days. **(A)** Period-amplitude analysis was performed for individual slow waves acquired with HD-EEG array during ZT1–3. Representative raw traces (top) and the corresponding downshifted EEG after subtracting the upper envelop of the trace (bottom). The detected slow waves are highlighted by increased line thickness. (**B**) A representative individual slow wave (black solid line) detected by the template-matching algorithm (red dashed line). ∆V is the peak-to-peak amplitude of the slow wave, and the falling and rising slopes were obtained by ∆V/τ_1_ and ∆V/τ_2_. (**C**) NREM sleep duration during ZT1–3 (mean ± SD). The asterisks represent a significant difference from the baseline value (*n* = 9; paired t-test; *p* < 0.05). (**D–H**) Total number (**D**), occurrence rate (**E**), mean amplitude (**F**), falling slope (**G**) and rising slope (**H**) of slow waves in anterior (channels #1–10) and posterior (channels #13–26) regions.

To investigate the influence of CSR on the occurrence of slow waves as well as its wave characteristics, individual slow waves were detected using template-based algorithm (see Methods) in the time traces of frontal and posterior EEG obtained from HD-EEG during ZT1–3 (Fig. 7A). While total duration of NREM sleep during ZT1–3 was progressively decreased from SR1 to SR5 (Fig. 7C), the total number of slow waves was moderately increased on SR1 followed by progressive decreases on SR 3 and 5 (Fig. 7D). To scale the intensity level of slow waves, the occurrence rate was calculated by dividing the number of slow waves by the NREM duration. The occurrence rate of slow waves was elevated significantly on SR 1, 2, and 3 and returned to the BL level in both anterior (channels #1–10) and posterior (channels #13–26) regions on recovery sleep days 1 and 3 (Fig. 7E). The peak-to-peak amplitude of slow waves increased significantly on all SR days (Fig. 7F). Following the first 18-h sleep deprivation (SR1), the amplitude of slow waves increased by 12.8% and 10.5% per day over SR days in the anterior and posterior regions, respectively. As shown in the representative trace of Fig. 7B, the falling and rising slopes of the negative deflection in EEG were calculated in terms of voltage per second (Fig. 7G,H). The magnitude of rising slopes on baseline as well as sleep restriction days was always greater than that of falling slopes (Fig. 7G,H; Supplementary Fig. S2). Following the first 18-h sleep deprivation (SR1), the magnitude of falling slope was increased by 6.2% in the anterior and 7.2% in the posterior region, followed by 2.5% and 2.2% daily increase from SR1 to SR5 in the anterior (*p* < 0.001, linear regression, R^2^ = 0.137) and posterior region (*p* = 0.008, linear regression, R^2^ = 0.137), respectively. Likewise, the rising slope was increased by 12.3% in the anterior and 3.9% in the posterior region on SR1. However, daily increases from SR1 to SR5 failed to reach statistical significance by linear regression analysis in both the anterior (*p* = 0.057) and posterior region (*p* = 0.119). Both the falling and rising slopes in the occipital area were not changed significantly from SR1 to SR5. These significant increases in the amplitude and the slopes during CSR returned to the BL levels on the first day of recovery.

## Discussion

**Figure 8:**
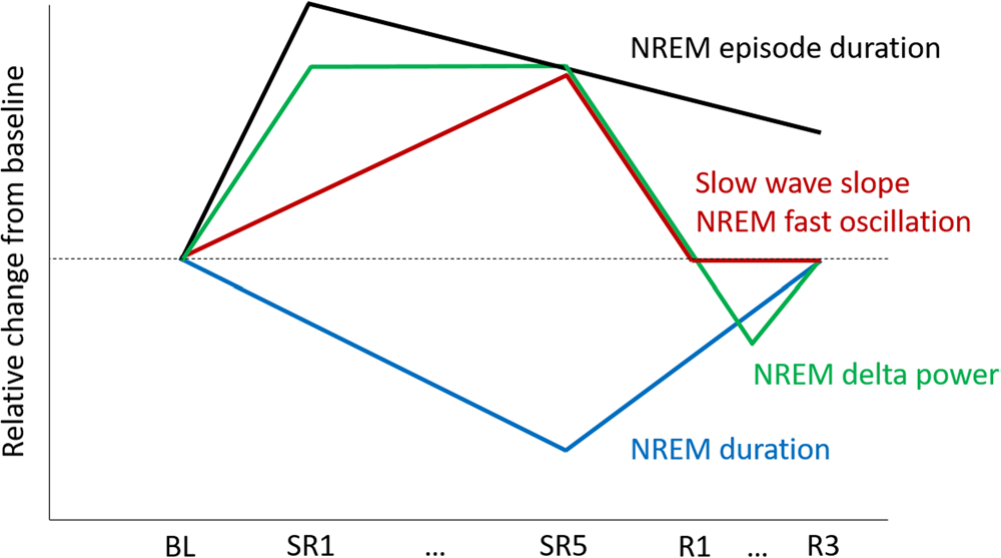
Summary diagram representing significant changes in sleep architecture during CSR in mice.

As summarized in Fig. 8, this study demonstrates that when daily 18-h sleep deprivation was repeated for 5 days, mice generated progressively less NREM sleep during the daily 6-h SO over the SR days we measured. NREM delta power during the first half of the 6-h SO was maintained at the elevated level on the SR days 1, 3 and 5. However, the period-amplitude analysis of NREM slow waves at the same period indicates that the frontal and posterior region of the cortex exhibited progressively increased slow wave slope. In addition, high frequency oscillation power in NREM sleep also showed progressive increases on the SR days. These results suggest that multiple sleep-wake regulatory systems exist in the brain, which can be operated independently, especially in the CSR condition.

### NREM sleep reduction is more robust in mice than in rats or humans

Previous reports comparing sleep phenotypes in different strains of mice suggest that the mouse exhibits genotype-specific sleep duration and EEG power profile^20^,^21^. However, almost all mouse strains exhibited increases in NREM delta power following spontaneous wakefulness or sleep deprivation^20^. In this study, we investigated one of the most commonly used hybrid strains for genetic studies (C57BL/6 J (B6) × 129/SvJ (SvJ)) and focused on analyzing homeostatic sleep responses to CSR condition.

One of the most striking findings is robust decreases in NREM sleep duration during sleep opportunities (Fig. 1). To compare the changes in NREM sleep duration between rats and mice in response to CSR, a meta-analysis was conducted between the results of the current study and our previous report using Sprague-Dawley rats^3^. Both studies used the same protocol: 18-h sleep deprivation (ZT6–24) followed by 6-h SO, which was repeated for 5 days. The relative reductions in NREM sleep duration on the last SR day (SR5) was much greater in mice than in rats (−36.6% vs. −21.8%, *p* = 0.032; see Supplementary Fig. S1 top panel). During recovery sleep days, rats showed an significantly elevated level of NREM sleep on the first recovery day (R1) while mice’s NREM sleep duration was still far below the baseline level (+8.7% vs. −18.6%, *p* = 0.001). Furthermore, the NREM sleep duration was completely returned to the BL level in rats by recovery sleep day 3, but in mice it was still below the BL level even though the difference was not statistically significant (−11.6%, *p* = 0.061).

Other rat studies have also reported a similar trend that compensatory increases in NREM sleep time during sleep opportunities are progressively reduced from SR day 1 to 5^4,11^. However, this pattern was not observed in one rat CSR study that used a repeated cycle of 3-h sleep deprivation and 1-h SO for 4 days^6^, and was also not clearly seen in human CSR studies^22,23^. Even though this study may need following investigations in different strains of mice, CSR-induced alterations in homeostatic NREM sleep duration seem a common feature at least in rodents.

### NREM delta power was persistently increased only immediately after daily sleep deprivation

Borbély’s Two-process model suggests that the magnitude of initial NREM delta power increase is correlated with the duration of previous waking^1^. Therefore, a longer period of sleep deprivation induces greater increases in initial NREM delta power. For the B6 x SvJ hybrid mice used in this study, however, we did not observe any statistically significant increase in NREM delta power during the 6-h SO following 18-h sleep deprivation or 5 days of SR (Fig. 4 top panel). This is in contrast to findings in rat CSR studies where significant increases in NREM delta power were reported at least on the first SR day^2,3^ or throughout all 5 SR days^11^. In the present study, we observed that NREM delta power was significantly increased only during the first 3 h of 6-h SO (see Fig. 4 second panel and Fig. 6D), and was not progressively increased despite of accumulated sleep loss as daily SR was repeated. Furthermore, this significant increase might be in part due to low baseline levels. As shown in Fig. 3, the baseline NREM delta power of the B6 x SvJ hybrid mice was lowest at the beginning of the light period (ZT0–4) and stayed at the highest level in the middle of the dark period. This pattern of delta power change in B6 x SvJ seems to stem from the sleep phenotype of its parental strain SvJ. For example, the peak NREM delta power occurred in the middle of the dark period (ZT18) in SvJ, while it was at the first hour of the light period (ZT0–1) in B6^20^.

### CSR-induced increase in NREM delta power was greater in the frontal cortex

We observed that baseline NREM delta power was higher in the frontal area than in any other cortical areas on the baseline sleep day (Fig. 6B). This is consistent with the observations in humans^13,24–26^. In addition, human HD-EEG studies also reported that total sleep deprivation for 1 night induced significant increases in NREM delta power, more robustly in the frontal area^25^. We also observed greatest increases in NREM delta power in the frontal area than parietal or occipital area (Fig. 6B,D). Our observations are also in agreement with the previous mouse^27^ and rat studies^11,28^ in which NREM delta power rebound following sleep deprivation or restriction was greater in the frontal cortex than in the occipital cortex.

The limitations of current mouse HD-EEG method used in this study includes (i) partial recording time due to the DC drift (see Methods for detail), which prevented collecting EEG continuously for the full 6-h SO period, and (ii) motion-related EEG artifacts, which made it difficult to analyze low frequency EEG signals (i.e., delta and theta power) especially during wakefulness. However, the intrusion of delta power into periods of waking during the sleep deprivation has been reported minimal in previous rat CSR studies^2,3,5,6^. The recent mouse CSR study also revealed that a little delta leakage during sleep deprivation is not sufficient to compensate significant NREM delta power reduction observed during sleep opportunities^7^.

### Slope of slow waves was progressively increased during CSR

Previous reports using period-amplitude analysis of slow waves in humans^29^ and rats^30^ showed that (i) occurrence rate of slow waves “with higher amplitude” or (ii) slow waves with steeper slope are positively correlated with the magnitude of NREM delta power. However, the occurrence rate of “total” slow waves regardless of the amplitude was not proportional to the NREM delta power. In contrast, our analysis implies that NREM delta power (Fig. 6D) may be positively correlated with most parameters of “total” slow waves including occurrence rate, amplitude and slopes in most brain areas examined (Fig. 7E–H).

Through the period-amplitude analysis from the local field potential (LFP) data collected from the rat frontal cortex^30^ and the accompanied computer modeling^31^, Tononi and colleagues argued that the rising slope in EEG (i.e., falling slope in LFP) indicates recruitment rate of neurons into the up state while the falling slope in EEG (i.e., rising slope in LFP) indicates a decruitment rate of neurons into the down state. Therefore, the greater magnitude of rising slopes compared to that of the falling slopes observed in the present study (Fig. 7G,H, Supplementary Fig. S2) and previous studies^30,31^ may indicate cortical neurons are faster in transitioning to the up state synchronously than to the down state. In addition, because we observed both falling and rising slopes were continuously increased as daily sleep restriction repeated, it seems that when net sleep losses are accumulated over days, the brain “accelerates” transition to up or down states, probably to dissipate the elevated sleep pressure more efficiently. Therefore, changes in slopes of individual slow waves in NREM sleep may be a critical indicator of sleep homeostasis, which cannot be assessed by power spectrum analysis that has been most widely used in sleep homeostasis studies. In addition, it would be interesting to investigate if the progressive increases of slow wave slope are associated with elevating sleepiness and diminishing neurocognitive performance in CSR conditions.

## Methods

### Animals

F1 male mice of C57BL/6 and 129S4/SvJae hybrid (12 weeks old at the time of surgery, *n* = 9) were used. Animals were maintained on a 12-h light: 12-h dark cycle (lights on at 8:00 AM) with freely available food and water. All animal experimental procedures were approved by the Institutional Animal Care and Use Committee at Korea Institute of Science and Technology, and were in accordance with National Institutes of Health guidelines for the treatment of animals.

### Surgical procedures

Surgical procedures to implant EEG electrodes were described previously^14,32^. Briefly, animals were anesthetized with ketamine and xylazine cocktail (120 and 6 mg/kg, respectively) by intraperitoneal injection, and positioned on a stereotaxic apparatus. If necessary, supplemental anesthetic (a third of the original dose) was administered. All mice were implanted with both the HD-EEG microarray and conventional screw electrodes.

The HD-EEG microarray implanting procedure was previously described in detail with video demonstration^33^. Briefly, the skull was exposed and wiped with water-soaked cotton balls, and a 40 channel thin film electrode microarray was aligned on the line between bregma and lambda on the skull. The center of the 5th wing of the electrode array was aligned above bregma.

For conventional EEG recording, three screw electrodes (stainless tapping screw, 0.8 mm × 4.8 mm, Nitto Seiko Co., Japan) were implanted: (i) one above the frontal area (1.5 mm anterior and −2 mm lateral to bregma), (ii) one above the parietal area (2 mm posterior and 4 mm lateral), and (iii) one on the interparietal bone above the cerebellum used as both a reference and ground electrode (6 mm posterior and ±2 mm lateral)^14^. Two additional screws were implanted to support the implant. All electrodes were secured to the skull with dental cement (Vertex-Dental, Zeist, Netherlands). The total weight of the electrodes including the connector and dental cement was 1.6 ± 0.2 g. Immediately after surgery, the animals were individually housed in their home cages with ambient temperature maintained at 24 ± 1 °C.

### Recording procedures

EEG recording procedures were described previously^14^. Briefly, during 7 d of recovery from surgery, the animals were habituated to an acrylic recording cage (7.8 inch in diameter x 9.8 inch in height) in a light- and sound-proof Faraday chamber. The animals were also habituated to the tether and connectors at least 2 d before recording. Conventional screw EEG was recorded simultaneously with the HD-EEG recordings, using an analog amplifier (Grass Technologies, Warwick, RI, USA, QP511) and digitized with an analog-digital converter (Axon Digidata 1440 A, Molecular Devices, Sunnyvale, CA, USA). The EEG signals were digitized with 500 Hz sampling rate, high-pass filter at 0.3 Hz, low-pass filter at 100 Hz, and 60 Hz built-in notch filter. For each mouse, two differential EEG signals were recorded: (i) between the frontal cortex and the cerebellum and (ii) between the parietal cortex and the frontal cortex. The cables for screw EEG were connected to the head cap via a commutator (Plastics One, Roanoke, VA, USA).

For HD-EEG recording, a Synamps 2 amplifier and a SCAN 4.5 data acquisition system (CompuMedics USA, Charlotte, NC, USA) were used. The HD-EEG signals were recorded at 1 kHz sampling rate and band-pass filtered between 0.1 Hz and 100 Hz. All high-density recording channels were referenced to a posterior channel above the cerebellum area. The electrode in the microarray can load DC drift to accumulate charging current in the electrode-skull interface, leading to saturation of the EEG signals. Therefore, HD-EEG recording were limited to 3 h without a cessation.

For monitoring the mouse movement, a custom-designed motion sensor using an accelerometer (KXTC9–2050, Kionix Inc., Ithaca, NY, USA) was attached to the EEG connector^34^. Accelerometer signals were also digitally converted by the Axon Digidata 1440 A at sampling rate of 500 Hz.

### Sleep deprivation procedures

Sleep deprivation procedures were described previously^14,32^. Briefly, sleep deprivation was produced by the intermittent rotation of wheels (5.5 inch in diameter × 2.3 inch in width, Lafayette Instrument, Lafayette, IN, USA, #80860 modified) programmed on a repeating cycle of 4-s on (approximately 2.3 RPM) and 2-s off schedule during the daily 18-h periods of sleep deprivation. Each time, the wheel was rotated about one-sixth of a revolution preventing the mice from entering into sleep. Mice had free access to food and water during sleep deprivation periods. After 18-h sleep deprivation, animals were transferred to their home cages for the 6-h sleep opportunities.

### Data analysis

Conventional screw EEG data analysis procedures were described in detail elsewhere^2,3^. Briefly, EEG data of the frontal cortex (referenced to the cerebellum) were converted to ASCII format using Clampfit 10.2 (Molecular Devices, Sunnyvale, CA, USA). SleepSign software (Kissei Comtec Co., Japan) was used to manually score 10-s EEG epochs as wake, NREM sleep or REM sleep. Epochs containing EEG artifacts were less than 1% even on SR5, which were excluded from the power spectral analysis (0.0% on BL and 0.9 ± 1.3% on SR5). Depending on the particular analysis, wake, NREM sleep and REM sleep time, NREM episode duration and numbers as well as delta power, were determined in 1, 2, 6, 18 or 24-h time blocks.

HD-EEG data analysis procedures were described previously^14^. Briefly, the HD-EEG data were further analyzed separately with MATLAB (Mathworks, Inc. Natick, MA, USA). The HD-EEG signals were divided into NREM epochs based on the synchronized data from the manual scoring of the conventional EEG and the motion sensor. FFT was applied to each NREM epoch of the HD-EEG and FFT’s squared magnitudes between 1–4 Hz were averaged. The values of NREM delta power during ZT1–3 were averaged for each day in each mouse. Power ratio to baseline day for each sleep restriction and recovery day was computed as follows: Power ratio = (P_day_ – P_base_)/P_base_, where P_base_ and P_day_ are the averaged delta power of NREM epochs for baseline day and the day of comparison, respectively.

The channels with contact impedance higher than 1 MΩ were excluded in the analysis. The topographical mapping was represented by a contour plot on the brain surface which were rendered by ‘spm_surf’ function in SPM8 (Welcome Trust Centre for Neuroimaging, UCL, London, UK) using the mouse magnetic resonance microscopy atlas (downloadable in http://www.loni.usc.edu/). The fictitious points for the contour were estimated with a cubic spline interpolation method on the imaginary 2-D 250 × 250 meshgrid based on the coordinates of microarray.

Statistical comparisons of sleep-wake parameters across BL, SR1-SR5 and R1-R3 conditions were described previously^3^. Briefly, a repeated-measures analysis of variance (ANOVA) test was used for ZT0–6, ZT6–24, and 24-h total separately. Once there is a significant time effect, post-hoc comparisons between BL and other experimental days were made using the Fisher’s LSD test. For comparing 3 days of recovery sleep from the baseline sleep in 2-h intervals, paired two-tailed *t*-test was used. Comparisons were considered statistically significant if *p* < 0.05. All error bars in the figures and the ranges following the mean values in the text represent standard deviation.

### Detection and analysis of individual slow waves

Template matching is a technique used to isolate an event buried in complex signals by comparing the similarity between a predefined template and the possible events in the signals that closely resemble the template^35^. Following developing a template matching algorithm, detection of individual slow waves was performed on the frontal and parietal EEG, averaged over frontal (channels #1–10) and posterior regions (channels #13–26), respectively. To increase the detection rate, the EEG signals were digitally low pass-filtered (6 Hz cutoff, zero-phase shift, 6th-order Butterworth filter). The background slow fluctuations were also eliminated by an envelope-removal method, which acts as a high-pass filter without distorting the peak locations (Fig. 7A). Specifically, the upper envelope was obtained by tracking the successive positive peaks of the filtered signal and then connecting these points by a cubic spline interpolation. Then the EEG signals were down-shifted to the zero line by subtracting the upper envelope of the signals. EEG segments, spanning between two neighboring zero-crossings of a negative peak, were selected and then an adaptable template with an asymmetric triangular waveform was created in a way that the triangular points match to the negative peak and the zero-crossing points (Fig. 7B). The slow wave events were detected by calculating the cross-correlation coefficient between the EEG segment and the template with a threshold of 0.7 (see Fig. 7A). The segments shorter than 120 ms and longer than 700 ms were excluded in the template matching test, thereby selecting waves of approximately 0.7 ~ 4.2 Hz. Detecting waves at this period range best correlated with the arousal state of each visually scored epoch, even though a conventional delta range (i.e., 1~4 Hz) was used for the power spectral analysis of HD-EEG data.

Data were compared using paired Student’s t-test when appropriate. To test a time effect over different experimental days, repeated- measure one-way ANOVA was used. For all tests, *p* < 0.05 was considered statistically significant.

## Supplementary information

Supplementary information 
