# Supplementary material for: Differential modulation of NREM sleep regulation and EEG topography by chronic sleep restriction in mice
Source: Sci Rep. 2020 Jan 10;10:18. doi: 10.1038/s41598-019-54790-y (PMC6954245; doi:10.1038/s41598-019-54790-y)
Supplement: Supplementary file 1 — Supplementary information [file 41598_2019_54790_MOESM1_ESM.pdf]

Supplementary Information

Title: Differential modulation of NREM sleep regulation and EEG topography by chronic sleep restriction in mice

Authors and Author Addresses: Bowon Kim[1, 2], Eunjin Hwang[1], and Robert E. Strecker[4], Jee Hyun Choi[1, 3], Youngsoo Kim[4]

1. Korea Institute of Science and Technology, Center for Neuroscience, Seoul, South Korea
2. Yonsei University, Medical Science Department, Seoul, South Korea
3. University of Science and Technology, Department of Neuroscience, Daejon, South Korea
4. VA Boston Healthcare System, Research Service and Harvard Medical School, Department of Psychiatry, Brockton, MA, USA

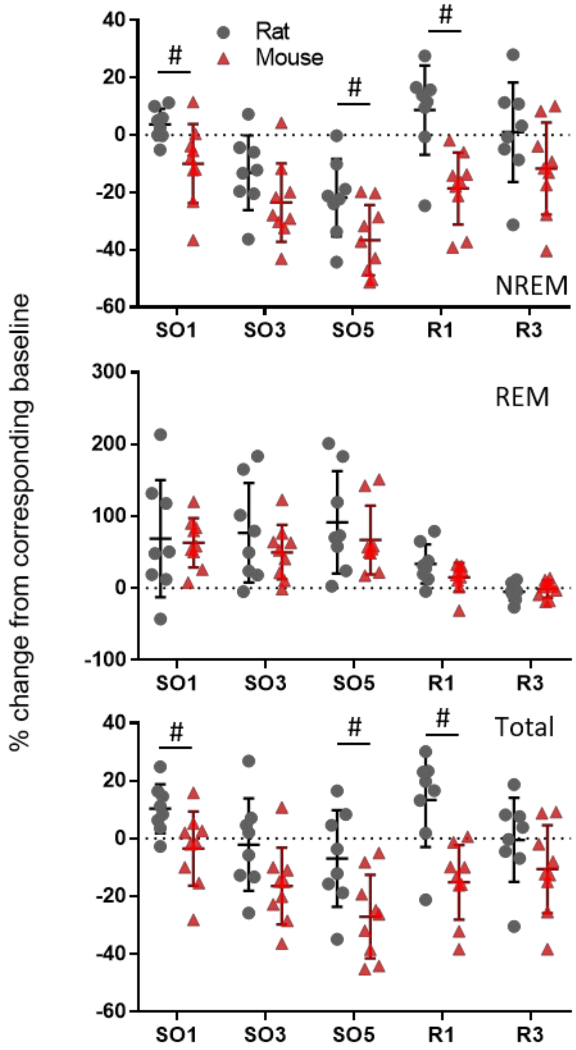

Supplementary Figure S1. Mice sleep less than rats during chronic sleep restriction. The duration (mean ± SD) of NREM (top panels), REM (middle panels), and total (bottom panels) sleep was determined during 6-h sleep opportunities (SO) and 24-h recovery sleep (R) periods in rats (Sprague-Dawley) and mice (hybrid of C57BL/6J x 129/SvJ) which underwent 18-h sleep deprivation for 5 consecutive days. Overall, NREM and total sleep of mice were more reduced than those of rats during 6-h SO over 5 days of sleep restriction. On recovery sleep days, both NREM and REM sleep time in rats were increased on R1 and returned to the baseline level on R3. In contrast, NREM sleep time in mice was still in a reduced level until R3. Rat sleep data were reanalyzed from our previously published study that used an identical protocol [1]. Statistically

significant difference ($p < 0.05$) was indicated by the pound sign (#) for comparison between rats and mice (n = 8 for rat and 9 for mouse, Student *t*-test).

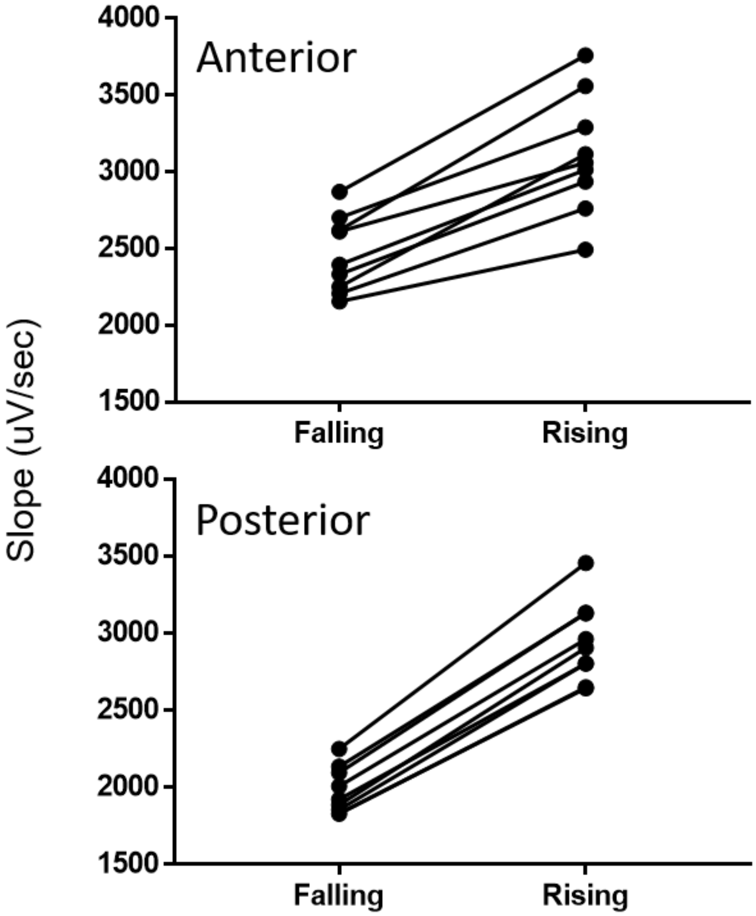

Supplementary Figure S2. Rising slopes of individual slow waves are steeper than falling slopes. Baseline slopes were plotted by different brain regions (*n* = 9 each). Paired t-test indicates that rising slope is greater in both frontal ($p < 0.0001$) and posterior regions ($p < 0.0001$).

Reference for Supplementary Information

1 Kim, Y. *et al.* Decoupling of Sleepiness from Sleep Time and Intensity during Chronic Sleep Restriction: Evidence for a Role of the Adenosine System. *Sleep* **35**, 861-869, doi: 10.5665/sleep.1890 (2012).
